# Response and inbreeding from a genomic selection experiment in layer chickens

**DOI:** 10.1186/s12711-015-0133-5

**Published:** 2015-07-07

**Authors:** Anna Wolc, Honghua H. Zhao, Jesus Arango, Petek Settar, Janet E. Fulton, Neil P. O’Sullivan, Rudolf Preisinger, Chris Stricker, David Habier, Rohan L. Fernando, Dorian J. Garrick, Susan J. Lamont, Jack C. M. Dekkers

**Affiliations:** Department of Animal Science, Iowa State University, Ames, IA 50011-3150 USA; Hy-Line International, Dallas Center, IA 50063 USA; Lohmann Tierzucht GmbH, 27472 Cuxhaven, Germany; agn Genetics GmbH, Börtjistrasse 8b, 7260 Davos, Switzerland

## Abstract

**Background:**

Genomic selection (GS) using estimated breeding values (GS-EBV) based on dense marker data is a promising approach for genetic improvement. A simulation study was undertaken to illustrate the opportunities offered by GS for designing breeding programs. It consisted of a selection program for a sex-limited trait in layer chickens, which was developed by deterministic predictions under different scenarios. Later, one of the possible schemes was implemented in a real population of layer chicken.

**Methods:**

In the simulation, the aim was to double the response to selection per year by reducing the generation interval by 50 %, while maintaining the same rate of inbreeding per year. We found that GS with retraining could achieve the set objectives while requiring 75 % fewer reared birds and 82 % fewer phenotyped birds per year. A multi-trait GS scenario was subsequently implemented in a real population of brown egg laying hens. The population was split into two sub-lines, one was submitted to conventional phenotypic selection, and one was selected based on genomic prediction. At the end of the 3-year experiment, the two sub-lines were compared for multiple performance traits that are relevant for commercial egg production.

**Results:**

Birds that were selected based on genomic prediction outperformed those that were submitted to conventional selection for most of the 16 traits that were included in the index used for selection. However, although the two programs were designed to achieve the same rate of inbreeding per year, the realized inbreeding per year assessed from pedigree was higher in the genomic selected line than in the conventionally selected line.

**Conclusions:**

The results demonstrate that GS is a promising alternative to conventional breeding for genetic improvement of layer chickens.

## Background

Genomic selection (GS) as a means of marker-assisted improvement was introduced by Meuwissen et al. [1] and has been implemented for dairy cattle [2], among other species. Genomic prediction of merit requires a training population that includes genotyped individuals with individual or offspring phenotypes. Animals are genotyped with a large number of markers (typically more than 10 000 single nucleotide polymorphisms, SNPs) that are located across the genome. Training data are used to develop a model to predict breeding values based on SNP genotypes, and this model is used to predict breeding values in future generations [1]. One of the main challenges of implementing GS in breeding programs for poultry is the large number of selection candidates and the limited value of individual candidates compared to the cost of genotyping. Nevertheless, using deterministic simulation, Sitzenstock et al. [3] recently demonstrated that implementation of GS in breeding programs in layer chickens can result in extra genetic and economic gains, in particular when the breeding program is redesigned to capitalize on the ability of GS to reduce the generation interval.

Although empirical accuracy of genomic prediction has been studied in most livestock species (dairy and beef cattle, swine, broiler and layer chickens), studies on the impact of empirical results of GS on genetic gain are not available. In addition to increasing accuracy of selection at young ages, GS is expected to reduce rates of inbreeding per generation because GS provides additional information on Mendelian sampling terms of selection candidates [4, 5]. GS is predicted to allow a reduction in size of breeding programs, without increasing the rate of inbreeding. The reduced costs in management and performance recording for a smaller population size should help offset the considerable investment in genotyping that is required. Alternative or additional strategies could also involve preselection of candidates for genotyping.

The objectives of this study were to design, simulate, implement and retrospectively evaluate a GS program for layer chickens, which could potentially double the response to selection per year, while maintaining the same annual rate of inbreeding, compared to a typical pedigree and performance-based layer selection program. The selection lines for the GS and conventional breeding programs were derived from the same foundation line, and multiple-trait performance was compared over the same time period in the terminal generations.

## Methods

This research was conducted in four steps as follows:

1. Design of a genomic selection program for layer chickens using deterministic prediction models based on selection theory that would double the response to selection per year but maintain the same rate of inbreeding per year compared to a conventional selection program based on pedigree and phenotypic information. Our aim was to achieve these goals while minimizing genotyping and phenotyping costs by changing the population structure.
2. Evaluation of the performance of this GS strategy by stochastic simulation.
3. Implementation of the GS and conventional breeding strategies, in which both selection lines were derived from the same foundation generation of a real elite purebred layer chicken population.
4. Retrospective evaluation of the realized responses to selection for the two breeding programs.

### Design of the genomic selection strategy using deterministic prediction

Responses to selection and rates of inbreeding were predicted by deterministic methods for a conventional BLUP (best linear unbiased prediction) selection program and for a range of GS programs in order to identify a strategy that could double the response to selection per year with the same rate of inbreeding per year as the conventional program. To reflect selection for egg laying traits, we considered a trait with a heritability of 0.3 and phenotypes available only on females at 1 year of age. The program SelAction [6] and procedures of [5] were used to predict asymptotic rates of response and inbreeding for the conventional BLUP and GS strategies.

The conventional BLUP strategy assumed selection and mating of 60 males and 360 females per 56-week long generation cycle. The 60 males were selected from 1080 selection candidates (three sons raised per hen) based on their BLUP estimated breeding values (EBV) from phenotypic data on female ancestors and sibs (individual phenotypes or progeny with phenotypes were not available at the time of selection). The 360 females were selected from 2880 selection candidates (eight daughters per hen) based on BLUP EBV from the phenotypes of female ancestors and sibs, as well as individual phenotypes. Deterministic predictions of this program using SelAction showed an expected response of 0.48 phenotypic standard deviation per generation and a rate of inbreeding of 1.38 % per generation.

Using the response to selection and rate of inbreeding of the conventional program as targets, predicted responses and rates of inbreeding per generation were evaluated for a large number of GS strategies; the number of sires selected was varied from 25 to 60, the number of dams selected from 40 to 120, and the number of male and female offspring per dam that were genotyped was varied from 3 to 12. The initial accuracy of genomic EBV that were assumed to be available on all selection candidates was set equal to 0.7, which resulted in an asymptotic accuracy after accounting for the Bulmer effect between 0.58 and 0.60 for all GS strategies. Based on deterministic predictions, a GS breeding program with 50 males and 50 females that were selected at each generation from 300 selection candidates per sex (six male and six female progeny from each single sire-dam mating) was predicted to result in a similar rate of response per generation as the conventional BLUP program (0.43 vs. 0.48 phenotypic SD), with less than half the rate of inbreeding per 28-week generation cycle (0.59 versus 1.38 %), while minimizing the number of individuals genotyped per generation. Thus, with the generation interval for the GS program being half that of the conventional 56-week program, this GS program was predicted to nearly double the response to selection per year, with a slightly lower rate of inbreeding per year.

### Evaluation of the genomic selection strategy by stochastic simulation

The conventional and GS programs described above were compared by stochastic simulation to validate these deterministic predictions. A genome that comprised 20 chromosomes of 37.5 cM each, for a total of 750 cM, was simulated. In the foundation generation of the base population (g0), 6001 equally spaced SNPs per chromosome were simulated for 500 individuals, with allele frequencies of 0.5 and in linkage and Hardy-Weinberg equilibrium. To generate mutation-drift equilibrium, the subsequent 1000 generations (g1-g1000) were simulated with random mating, mutation (rate = 2.5*10^−5^), and recombination, with effective population sizes of 500 for g1-g900 and 100 for g901-g1000. To create the training data, the population was expanded to 1000 individuals in g1001. Breeding values were generated by designating 200 random segregating (minor allele frequency ≥ 0.1) SNPs as quantitative trait loci (QTL). Each QTL was assigned an effect that was drawn from a Gamma distribution with shape parameter 0.4 and inverse scale parameter 1.66, following Hayes and Goddard [7]. The QTL effects were scaled such that the genetic variance in g1001 was 3/7. Each phenotype was simulated by adding a random environmental effect drawn from a standard normal distribution, assuming heritability to be equal to 0.3. Another 6000 segregating SNPs across the whole genome were sampled in the same base population and used as markers starting in g1001, which resulted in an average of eight SNPs per cM and is equivalent to 24 000 segregating SNPs for typical livestock genomes. These SNPs were used for training the genomic prediction models to reflect the real situation in which it is likely that causal mutations are not included in the SNP panels but markers with different levels of LD with the causal loci are. A total of 1000 females with phenotypic data were available to provide training data in g1001.

Starting in g1002, the conventional phenotype-based BLUP selection program (generation interval of one year) and the GS program (generation interval of 0.5 year) were simulated. Response and rate of inbreeding were evaluated over four generations of conventional selection and eight generations of GS because the generation interval was reduced by 50 % in GS.

For conventional selection, EBV were estimated at each generation by fitting the conventional animal model to available phenotypic data [8, 9], using a numerator relationship matrix based on pedigree going back to g1001, with generation as a fixed effect. Heritability was set equal to the true heritability of 0.3 in the base population. For GS, the BayesB method of [1] was used, by fitting the following model to the phenotype of individual i in the training data:

$$ {\mathrm{y}}_{\mathrm{i}}=\upmu +{\displaystyle {\sum}_j{\mathrm{X}}_{\mathrm{i}\mathrm{j}}{\mathrm{g}}_{\mathrm{j}} + {\mathrm{e}}_{\mathrm{i}},} $$

where μ is the generation effect, summation Σ_j_ is over all genotyped SNPs, X_ij_ is the number (0, 1 or 2) of copies of allele 1 that individual i carries at SNP j, g_j_ is the allele substitution effect for SNP j, and e_i_ is a random residual. Allele substitution effects g_j_ were assumed to be normally distributed with mean 0 and variance σ_gj_^2^ with probability 1-π = 0.05 or to be null with probability π = 0.95. Preliminary analyses found that the choice of π had a limited impact on the results. The prior for the variance of the substitution effects σ_gj_^2^ was χ^−2^(4.234, 0.0429). Estimates of allele substitution effects for each SNP, *ĝ*_*j*_, were obtained as posterior means from 1000  cycles of a Gibbs chain, of which the first 100 cycles were discarded as burn-in, combined with 10 cycles of a Metropolis-Hastings algorithm within each Gibbs cycle to obtain samples of σ_gj_^2^. Preliminary analyses showed that these numbers of cycles led to converged estimates of EBV. For selection candidates, EBV were computed based on their SNP genotypes as:

$$ {\mathrm{EBV}}_{\mathrm{i}}={\displaystyle {\sum}_j{X}_{ij}{\widehat{g}}_j} $$

GS was evaluated with and without retraining. Without retraining, the SNP effects that were estimated based on g1001 were used for all eight cycles of selection. With retraining, SNP effects were re-estimated at each generation, after adding phenotypes of the 300 female candidates from the previous generation to the training dataset.

Responses to selection were evaluated based on the mean true breeding value of selection candidates at each generation based on 48 replicates of the simulation. The level of inbreeding at each generation was based on the average pedigree-based inbreeding coefficients of selection candidates, using g1001 as the founder generation. Standard deviations of response and inbreeding were evaluated across the 48 replicates.

### Implementation of genomic selection

Based on the simulation results, a GS program was implemented in an experimental pure-bred commercial brown egg layer chicken line that was previously under conventional BLUP selection. To allow side-by-side comparison of the conventional and GS programs, the line was split into two sub-lines (later denoted as genomic and pedigree lines) by random splitting of full-sib families. The pedigree line underwent two subsequent generations of conventional selection, whereas the genomic line underwent four generations of GS. All birds were produced, reared, and managed in facilities of the Hy-Line breeding program by Hy-Line staff, using standard protocols implemented by Hy-Line International.

### Population structure and selection strategy

**Fig. 1:**
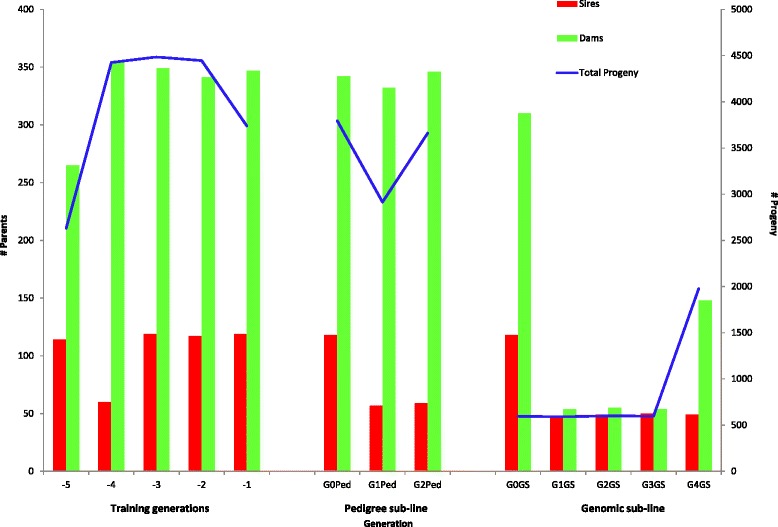
Numbers of contributing sires and dams and total number of selection candidates in the experimental breeding program, including training generations, three generations of the pedigree sub-line and five generations of the genomic sub-line

Different population structures were implemented for the two sub-lines, with numbers of contributing sires and dams and the total number of selection candidates shown on Fig. 1. In the pedigree line, 1000 male and 3000 female candidates were produced at each generation from 60 male and 360 female parents that were selected on a multi-trait index of phenotype-BLUP EBV, after records on female candidates were collected, with a restriction on the number of full-sibs selected. Selected males and females were mated in a hierarchical manner (six females per male), with some restriction to avoid matings between full- or half-sibs.

The GS program that was implemented was a slight modification of the one used in the simulation because it applied two-stage female selection. No changes in selection strategy were implemented on the male side: 50 males were selected at a young age on a multi-trait index of genomic EBV (GEBV). In the first stage of female selection, 150 of the 300 female candidates were selected at a young age based on a multi-trait index of GEBV. All 150 females were mated to produce progeny for the next generation and before being recorded for phenotypes, which resulted in a generation interval of 28 weeks. A partially cross-classified mating design was implemented to reduce rates of inbreeding and capitalize on the ability of the GS procedure to assign parentage based on genotypes. For this mating design, the 50 selected males and 150 females were divided into five mating groups of 10 males and 30 females. Within each mating group, each male was mated to a different set of three females by artificial insemination. Males were rotated between the sets of females within a mating group. Parentage of the chicks was established based on SNP genotypes. In the second stage, at 42 to 46 weeks of age, after most individual phenotypes had been recorded, the 150 females were re-evaluated based on a combination of GEBV and own performance. From the best 50 dams 300 male and 300 female progeny (now 14 weeks of age) were selected for genotyping and phenotyping. The remaining progeny were kept only for phenotyping. This two-stage selection strategy made it possible to reduce the generation interval by 50 %, while increasing the accuracy of female selection, although at the cost of doubling the number of progeny that were produced and reared.

Using progeny that could be uniquely assigned to the parents and excluding animals which had no progeny, each sire had on average 12.4 progeny (ranging from 1 to 31) and each dam 10.8 progeny (ranging from 1 to 15), with 1.9 (ranging from 1 to 7) full-sib progeny on average per sire-dam combination. The numbers of selection candidates, and final numbers of contributing sires and dams are in Fig. 1. Generation 3 of GS (G3GS) had additional phenotypes from non-genotyped progeny.

### Selection criteria

All phenotypes used in this study were obtained from routine data collection of Hy-Line International. Both sub-lines were selected based on the same multi-trait index combining 16 production and quality traits measured at early (e) or later (l) ages: age of sexual maturity (eSM, d), body weight at late age (lBW,g), shell color (based on an index obtained from the l, a, b Minolta® Colormeter system) for the first three eggs (eC3), at early (eCO) and late age (lCO), egg weight (g) for the first three eggs (eE3), at early (eEW) and late age (lEW), puncture score at early (ePS) and late age (lPS), albumen height (mm) at early (eAH) and late age (lAH), and yolk weight (g) at early (eYW) and late age (lYW). Egg production was expressed as ePD and lPD, which are the egg production rates (ratio of the number of saleable eggs to number of days in lay). Early measurements for egg quality traits were taken at 26 to 28 weeks of age. Late measurements for egg quality traits were taken at 42 to 46 weeks of age on birds not culled after early measurements. In addition, egg number (eEN and lEN, which are the total numbers of eggs (regardless of saleability) laid in weeks 1 to 10 and in weeks 11 to 20 of production, respectively) were monitored for correlated response. Complete records for late egg production were not available on GS females at the final stage of selection. Early and late egg quality measurements were averages of three to five eggs. Observations that deviated from the within-hatch generation mean by more than three standard deviations were excluded from breeding value estimation and treated as outliers, but all biologically feasible values were retained for line comparison in the final generation.

Breeding values of selection candidates in the pedigree sub-line were estimated using the pedigree-based multi-trait BLUP that is used for routine evaluation in the Hy-Line International breeding programs. Hatch-by-generation was used as the only fixed effect to account for contemporary group effects.

Selection candidates in the genomic sub-line were genotyped using a custom high-density Illumina SNP panel which provided 23 356 segregating SNPs (minor allele frequency > 0.025; maximum proportion of missing genotypes < 0.05; maximum mismatch rate between parent-offspring pairs < 0.05; parentage probability > 0.95). The same panel was also used to establish the training population for the first generation, which consisted of all selected parents from the previous five generations: 2708 genotyped animals, of which 1563 were females with individual phenotypes and 1145 were males without phenotypes. In addition, phenotypes of 11 486 progeny of the genotyped individuals were included as progeny means in the training data. In subsequent generations, phenotypes and genotypes of the 300 genotyped female selection candidates were added to the training population, i.e., the retraining option for GS was used.

In the final generation, the GS line was expanded to produce enough progeny to make comparisons with the pedigree sub-line valid. A total of 2318 progeny were hatched, of which 1977 were assigned to 49 sires and 148 dams based on low-density SNP genotypes (71 SNPs). Animals that were not genotyped or that had one or more parents not matching the mating scheme were excluded.

At each generation, the accuracy of several genomic evaluation methods was evaluated by using phenotypes of the last available generation for validation and the method with the highest accuracy was used for training on a dataset that included the phenotypes of the last generation for each trait. The genomic evaluation methods that were used included: univariate and bivariate GBLUP (using the early and late phenotypes for a given trait), GBLUP with a modified genomic relationship matrix [10], univariate BayesA, BayesB, and BayesCPi [11]. BayesCpi and GBLUP tended to have the highest accuracy and were therefore the predominant methods used. Variance components were estimated from the data using a multi-trait animal model. Because not all individuals were genotyped, both individual performance and progeny means were used for genomic evaluation by applying methods described by [12].

Bayesian analyses were performed using the GenSel software [13], while conventional BLUP and GBLUP analyses, which estimated variance components simultaneously, were done using ASReml [14]. Because the scale of GEBV can differ from the scale of the observed phenotypes, the GEBV of each trait were rescaled at each generation by multiplying by the coefficient of regression of adjusted (for hatch effects) phenotypes on GEBV obtained from the latest validation analysis.

For the second stage selection of females, the rescaled first stage GEBV were combined with each hen’s adjusted own phenotypes, using a simple index of GEBV and own phenotype for each trait. The weights assigned to own phenotype (b_1_) and GEBV (b_2_) in this index were derived using selection index theory [5], based on the accuracy of GEBV obtained in the latest validation analysis (*r*) and the associated estimate of pedigree-based heritability (*h*^2^):

$$ \left[\begin{array}{c}\hfill {b}_1\hfill \\ {}\hfill {b}_2\hfill \end{array}\right] = {\left[\begin{array}{cc}\hfill 1\hfill & \hfill {r}^2{h}^2\hfill \\ {}\hfill {r}^2{h}^2\hfill & \hfill {r}^2{h}^2\hfill \end{array}\right]}^{-1}\left[\begin{array}{c}\hfill {h}^2\hfill \\ {}\hfill {r}^2{h}^2\hfill \end{array}\right]. $$

### Evaluation of response to selection

In the final generation of the experiment, the two sub-lines were hatched and housed together for a direct comparison of performance. Since the environmental conditions were the same, least square means for the line from a model including effects of line and hatch were assumed to reflect genetic differences resulting from the two methods of selection. Calculations were performed with SAS [15]. In order to have a reference point for response to selection since the start of the experiment, the data from the pedigree line were analyzed with a multi-trait pedigree-based animal model, with variance components estimated from the data, and response to selection in the pedigree line was estimated based on the difference in average EBV in the last and first generations of the experiment. Estimates of the line difference from the final generations were then added to responses to selection for the three generations in the pedigree line to obtain estimates of response to selection in the genomic line. Responses were expressed in genetic standard deviations.

### Evaluation of inbreeding rate

Pedigree-based inbreeding was evaluated in both lines using the CFC software [16]. For the genomic line, genomic measures of inbreeding were calculated using PLINK [17], including average homozygosity, number and size of homozygosity runs of at least 50 consecutive SNPs, and inbreeding coefficients based on expected vs. observed homozygosity.

## Results

### Simulation results

**Fig. 2:**
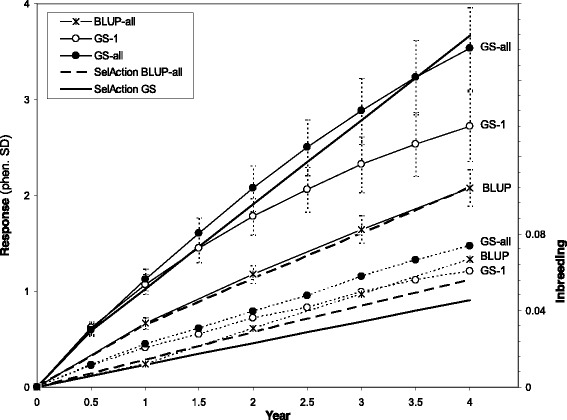
Expected responses to selection and inbreeding based on stochastic and deterministic simulation using conventional and genomic selection. Cumulative responses to selection (in phenotypic standard deviations, the scale on the left axis, top 5 lines) and inbreeding based on stochastic simulation (48 replicates, the scale on the right axis, bottom 5 lines) for conventional BLUP selection and genomic selection (GS) in layer chickens; GS-1 = GS with training on data from generation −1; GS-all = GS with retraining using data from all generations, up to but not including the current one; generation interval is 1 year for conventional BLUP and 0.5 years for GS; analytical predictions obtained from SelAction are also included; error bars are standard deviations of response across replicates

Observed average responses to selection and inbreeding rates based on stochastic simulation for the conventional and GS breeding programs are in Fig. 2. Results are shown on a per year basis and account for the fact that the generation interval for GS was reduced by 50 % compared to that of the conventional BLUP selection. The simulated conventional BLUP-based breeding program resulted in responses to selection that were similar to those predicted by SelAction. Two main scenarios are summarized in Fig. 2: genomic selection with retraining (GS-all) and without retraining (GS-1). For both GS-1 and GS-all, the accuracy of GS-EBV in year 0 (the generation following training) was equal to 0.77, which was slightly higher than the starting accuracy used to obtain deterministic predictions with the SelAction program (0.7). Accuracy in year 0 was the same for GS-1 and GS-all because retraining in a particular generation was done before females from that generation had their own performance records. Accuracy remained fairly constant for GS-all through year 2.5 (results not shown) and then gradually dropped to 0.73 by year 4. For GS-1, accuracy gradually dropped to 0.34 in year 4. Resulting responses to selection for GS-all were similar to those predicted by SelAction. For GS-1, observed responses were similar to those predicted by SelAction through year 1.5 but dropped off after that because of the decline in accuracy.

Consistent with the target, the rate of inbreeding on an annual basis was of the same order of magnitude for GS and BLUP approaches (Fig. 2). Observed rates of inbreeding were, however, greater than predicted by SelAction for all programs, but in particular for BLUP and GS-all. For BLUP, the observed rate of inbreeding was 1.95 % per year when ignoring the lower rate in year 1, compared to a prediction of 1.44 % by SelAction. For GS-all, the observed rate was equal to 0.89 % per generation compared to that of 0.58 % predicted by SelAction. For GS-1, the observed rate was equal to 0.69 % when the substantially higher rates in the first two generations were ignored, which was only marginally greater than the rate of 0.58 % predicted by SelAction. The higher rates for GS-all and for GS-1 in the first two generations are likely caused by the implicit prediction of pedigree when data from recent ancestors are used for training.

Standard deviations of the cumulative response across replicates increased over generations for all strategies as a result of drift (Fig. 2). Standard deviations of the response were similar for GS-1 and GS-all in the initial generations but increased slightly faster for GS-all than for GS-1 and reached 0.43 and 0.37 phenotypic standard deviation units for GS-all and for GS-1, respectively. When strategies were compared at the same generation of selection, the standard deviation of the response for BLUP was similar to that for GS-1, despite the much greater level of inbreeding at a given generation for BLUP compared to GS.

### Experimental results

Estimates of heritability and genetic correlations between traits using all data from the pedigree selected line are in Table 1. All traits, except PD and PS, had moderate to high heritability estimates. Estimates of genetic correlations between measurements of the same trait at two ages (early versus late) were high. Egg quality traits were in general positively correlated with each other but negatively correlated with egg production.

**Table 1:** Pedigree-based estimates of heritability and correlations^a^ between traits based on data from the pedigree sub-line

Trait^b^	eE3	eEW	lEW	eC3	eCO	lCO	eAH	lAH	eYW	lYW	ePS	lPS	ePD	lPD	lBW	eSM
eE3	**0.65**	0.87	0.83	0.10	0.10	0.05	0.41	0.37	0.42	0.42	0.12	0.10	−0.36	−0.23	0.43	0.38
eEW	0.40	**0.72**	0.96	0.09	0.08	0.04	0.40	0.33	0.56	0.55	0.20	0.13	−0.30	−0.20	0.49	0.13
lEW	0.34	0.48	**0.73**	0.13	0.12	0.09	0.37	0.30	0.56	0.65	0.21	0.22	−0.38	−0.31	0.50	0.17
eC3	0.06	0.00	0.00	**0.66**	0.81	0.74	0.15	0.08	0.07	0.12	−0.07	0.04	−0.15	−0.04	0.18	0.04
eCO	0.08	−0.01	−0.01	0.33	**0.71**	0.96	0.13	0.06	0.05	0.10	0.04	0.09	−0.11	−0.03	0.15	0.11
lCO	0.09	0.03	0.09	0.24	0.42	**0.70**	0.08	0.02	0.06	0.11	0.09	0.15	−0.12	−0.07	0.14	0.05
eAH	0.22	0.21	0.15	0.00	−0.01	0.00	**0.55**	0.94	0.02	0.04	−0.12	−0.10	−0.07	−0.01	0.11	0.11
lAH	0.20	0.22	0.18	0.01	0.04	0.04	0.34	**0.55**	−0.03	−0.02	−0.13	−0.07	−0.11	−0.04	0.05	0.12
eYW	0.06	0.45	0.06	−0.01	−0.01	−0.01	−0.06	−0.01	**0.48**	0.89	0.18	0.13	−0.09	−0.06	0.44	−0.04
lYW	0.03	0.05	0.46	0.04	0.02	0.05	−0.06	−0.04	0.17	**0.54**	0.19	0.22	−0.28	−0.27	0.52	0.05
ePS	0.04	0.13	0.06	−0.01	0.08	0.00	−0.08	0.01	0.05	0.01	**0.27**	0.82	−0.19	−0.25	−0.02	0.01
lPS	0.03	0.05	0.09	−0.01	0.04	0.15	−0.04	−0.04	0.00	0.00	0.09	**0.34**	−0.28	−0.38	−0.03	0.04
ePD	−0.07	0.01	−0.14	0.05	0.10	−0.01	−0.02	−0.06	0.07	−0.08	0.03	0.04	**0.36**	0.87	−0.21	−0.23
lPD	−0.02	−0.01	−0.10	0.03	0.03	0.01	−0.02	−0.06	0.03	−0.07	0.04	0.06	0.49	**0.39**	−0.14	−0.13
lBW	0.04	0.04	0.08	0.07	0.10	0.07	0.01	0.05	0.10	0.18	−0.03	0.01	0.00	0.06	**0.62**	0.04
eSM	0.42	0.09	0.10	0.02	0.10	0.06	0.11	0.06	−0.03	−0.03	0.04	0.02	0.01	0.00	0.01	**0.57**

^**a**^Estimates of heritability are on the diagonal, genetic correlations above the diagonal, and residual correlations below the diagonal

^b^Egg weight for first three eggs (*eE3*), at early (*eEW*) and late age (*lEW*), shell colour for first three eggs (*eC3*), at early (*eCO*) and late age (*lCO*), albumen height at early (*eAH*) and late age (*lAH*), yolk weight at early (*eYW*) and late age (*lYW*) puncture score at early (*ePS*) and late age (*lPS*), *ePD* and *lPD* are egg production rates (ratio of the number of saleable eggs to number of days in lay) body weight at late age (*lBW*), age at first egg (*eSM*)

**Fig. 3:**
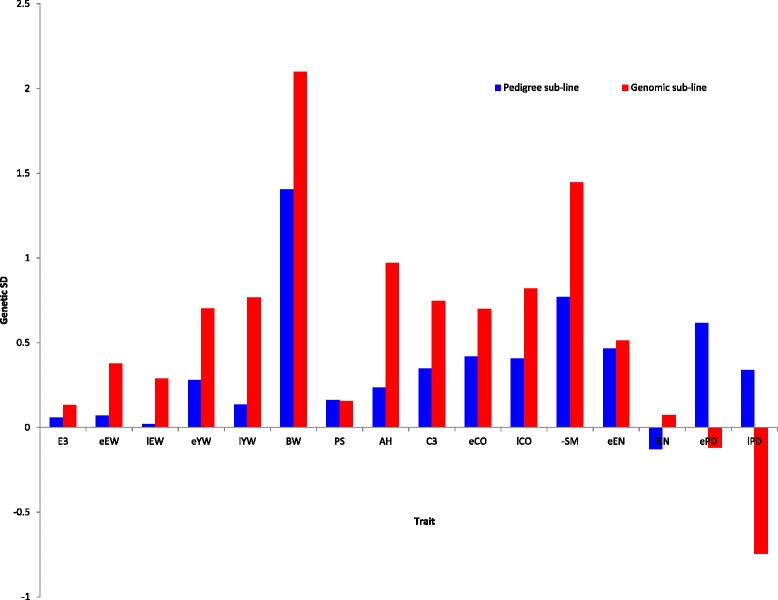
Responses to selection in the experimental breeding program, as deviation of trait means and expressed in genetic standard deviation units. Responses to selection are based on deviations of trait means from trait means at the start of the selection experiment, expressed in genetic standard deviation units of each trait; trait abbreviations: egg weight for first three eggs (eE3), at early (eEW) and late age (lEW), shell color for first three eggs (eC3), at early (eCO) and late age (lCO), albumen height at early age (eAH), yolk weight at early (eYW) and late age (lYW) puncture score at early age (ePS), egg production rates at early (ePD) and late (lPD), egg numbers at early (eEN) and late (lEN) age, body weight at late age (lBW), and age at first egg (eSM)

The standardized responses to selection by the end of the experiment are in Fig. 3. On average, trait means were changed in the desired direction by the end of the experiment for all traits. For most traits, the genomic line significantly outperformed the pedigree line, with a doubled response to selection for some traits, such as EW and YW. Body weight increased for both lines, with a larger response in the genomic line. This reflects selection for a revised objective, i.e., in the past, layer chicken lines were selected for lower BW, while, more recently, selection has aimed at increasing BW at a young age to allow pullets to develop adequately. For egg production rate (ePD and lPD), the pedigree selection line showed a positive response, while the genomic selection line showed a negative response to selection. It should be noted that, for this trait, phenotypes were available for the pedigree selection line at the time of selection but not for the genomic line. However, these results were not supported by the results of egg production measured by egg number (eEN and lEN), for which both lines showed a positive response, with a greater response for the genomic line. This difference in responses in egg production rate versus egg number was explained by both a higher frequency of egg defects in the genomic line (5.7 vs 4.0 %) and earlier age at sexual maturity of birds in the genomic line (141 vs. 145 days).

In the first 1.5 years of the selection experiment, inbreeding rate per year was similar between the two lines (Table 2), although the genomic line had twice as many generations and a smaller population size. However, in the final generation, the genomic line had a higher level of inbreeding than the pedigree line. The recorded pedigree of both lines traces back to 131 founders. In the pedigree line, all founders were represented in all three generations, with a variance of contributions in the final generation of 8.2E-05. In the genomic line, four of the founders were lost after six generations of selection but the variance of contributions at the end of the experiment was equal to 7.6E-05, which according to optimal contributions theory [18] should help to control inbreeding. At the end of the experiment, the most influential founder contributed 7 % of its genes to both lines.

**Table 2:** Molecular and pedigree-based estimates of inbreeding in the genomic sub-line

Generation	F_ped_	GenEq	NSEG	MB	MBAVG	F	AvHom
−5^a^	0.000	1	27.5	181.8	6.67	0.028	0.758
−4^a^	0.000	2	28.2	172.9	6.15	0.021	0.757
−3^a^	0.005	3	28.3	172.3	6.10	0.023	0.758
−2^a^	0.011	4	29.5	172.3	5.88	0.028	0.759
−1^a^	0.015	5	29.8	172.0	5.77	0.030	0.760
G0GS	0.018	6	29.8	159.1	5.34	0.023	0.758
G0Ped	0.019	6					
G1GS	0.020	7	31.1	156.9	5.03	0.023	0.758
G2GS	0.023	8	31.3	159.8	5.10	0.030	0.761
G1Ped	0.022	7					
G3GS	0.028	9	31.8	163.3	5.14	0.036	0.761
G4GS	0.041	10	33.0	170.4	5.17	0.055	0.766
G2Ped	0.026	8					

^a^Only selected parents were genotyped, *F*_*ped*_ average pedigree based inbreeding, *GenEq* average discrete generation equivalent, *NSEG* average number of segments with homozygosity runs longer than 50 consecutive SNPs, *MB* total length of homozygosity runs (Mb), *MBAVG* average length of homozygosity runs (Mb), *F* molecular inbreeding, *AvHom* average homozygosity

At the genomic level, the average homozygosity and number of homozygous segments increased over generations (Table 2), but the size of the homozygosity runs and genomic inbreeding dropped in generation G0, which was the first generation of genomic selection. Until generation G0, genomic inbreeding reflected the homozygosity level in the selected parents, in contrast to the following generations, in which all selection candidates were genotyped. The rate of inbreeding (based on pedigree and all genomic estimates) increased in generation 4 of the genomic line.

Genotypes were not available for the pedigree line but it was possible to compare changes in allele frequencies from generations −5 to 0 when pedigree selection was practiced with the changes observed with subsequent genomic selection. The average change in allele frequency was close to zero for both pedigree and genomic selected generations. The maximum average change in frequency per generation was equal to 0.062 for the pedigree selected generations and 0.097 for the genomic selected generations. Loci with the greatest changes (>5 SD) in allele frequencies were located on chromosomes 8, 9, and Z (six loci) for the pedigree selected generations and on chromosomes 6, 9, and 12 (four loci) for the genomic selection generations. Although the SNPs with the greatest change in frequency differed between the pedigree and genomic selected generations, several regions showed substantial changes in a consistent direction across the pedigree and genomic selected generations.

## Discussion

### Optimizing breeding programs with genomic selection

Use of marker information removes many of the limitations of conventional phenotype-based selection programs, as has been argued and demonstrated by many simulation studies [19], because phenotypic records on selection candidates and/or their close relatives are not required to estimate breeding values. This feature is even more pronounced for GS and provides opportunities to substantially change the structure of breeding programs [3, 20, 21].

In this study, we investigated the various opportunities that GS offers to improve breeding programs of layer chicken by changing their structure. Results clearly demonstrate that GS provides interesting opportunities to reduce generation intervals and the size of breeding programs, which impacts the number of animals that need to be raised and phenotyped on a routine basis. Maximizing response per year for a given rate of inbreeding per year was used as the objective for comparing alternative strategies for GS. This has been suggested as a reasonable objective for balancing short- and long-term responses to selection [22]. Even when reducing the generation interval by 50 %, the much lower rate of inbreeding per generation that resulted from GS because of the lower within-family correlations of EBV, allowed a substantial drop in the number of parents selected with GS for the same rate of inbreeding per year. Furthermore, with GS-EBV of equal accuracy for males and females and no limitations on reproductive rates, this resulted in equal numbers of selected males and females as being optimal. Removing the hierarchical mating restriction of one male per female that was used here in the simulation and allowing for factorial or cross-classified mating designs is expected to further reduce rates of inbreeding [23] or could reduce the number of parents needed for the same rate of inbreeding.

The simulated GS breeding program required much smaller numbers of selection candidates (500 males and 500 females per year for GS compared to 1080 males and 2880 females per year for the conventional program) to achieve rates of genetic gain per generation that approached those of the conventional program. This was due to the greater accuracy of EBV with GS, in particular for males, which had EBV based on pedigree and sibs in the conventional program. In addition, the numbers of individuals that were phenotyped were substantially smaller for the GS program, even with retraining (500 females per year for GS, compared to 2880 per year for the conventional program). Lower rearing, housing, and phenotyping requirements would substantially reduce the costs of breeding programs. Whether these reduced costs offset the extra costs associated with genotyping depends on specific prices. The parameters that we used for both the conventional and GS programs are given as examples to illustrate the opportunities provided by GS. Further analyses that consider specific prices, requirements and limitations are needed. The optimal strategy for retraining, including how often and on which animals new phenotypic data should be generated, also requires further investigation and will depend on the species used and the goals of the breeding program.

The GS program was successfully implemented, but the short generation interval was not very practical in a commercial breeding program setting. Reproducing females at a very young age increases selection pressure on sexual maturity, and requires using suboptimal sized eggs and hatching them over multiple hatches, which complicates optimization of animal management, especially the lighting program. When selection is initiated, the late maturing females have not started laying yet or produce eggs that are too small to hatch good quality chicks. A more feasible approach would be to use young genomic selected males on older females, which would also improve accuracy of selection because the females would already have own records. A novelty in the implemented GS breeding program was the use of cross-classified mating, in which females are given the opportunity to leave progeny from more than one male parent. Parent assignment with a high level of accuracy based on the number of opposing homozygotes in parent-offspring pairs was possible for almost all offspring from the multi-sire matings. The use of cross-classified mating improves population structure and creates more opportunities for varying chromosomal combinations in the progeny and thus, results in higher effective rates of recombination.

### Genomic prediction models

In the stochastic simulation, the Bayes-B method of [1] was used to develop the prediction model for GS. This method has been found to give greater accuracies than other methods in many simulation studies [1, 24]. The method was, however, not optimized with respect to the prior probability π that a SNP has zero effect; a value of π = 0.95 was used throughout, since preliminary analyses suggested that results were robust to choice of this parameter.

In the analyses on real data, we found only small differences between genomic evaluation models [25] and some variation in their ranking between generations, but on average they were consistently better than pedigree-based EBV. This lack of differences between methods is often interpreted as evidence that the number of QTL is large but could also result from genetic relationships and within-family effects having large impacts on GEBV [12]; in contrast to historic linkage disequilibrium, which is short-range, genetic relationships and within-family effects do not require markers that are close to the QTL and, therefore, the effects of QTL can be spread across markers surrounding the QTL.

### Impact of genomic selection on the initial accuracy of prediction

Analyses of real data showed that accumulation of information across generations improved the accuracy of genomic predictions on average [25]. In general, the trends observed with real data agree with those of the simulations but, for most traits, the accuracy of prediction estimated based on real data was lower than expected for a given heritability estimate, which suggests that the genetic architecture of traits is more complex than the simple additive model used in the simulations. This would also be in line with the poor consistency of estimates of small QTL over generations [26]. However, for some of the traits with a lower heritability, decreasing the weight on genomic information from very distant relatives of the selection candidates was shown to be advantageous [10]. Across traits, adding phenotypes from distant relatives (more than four generations apart from that of the selection candidates) did not improve the accuracy of predictions (Weng personal communication). Lourenco et al. [27] reported similar observations for cattle and pig data.

Previous analyses with this data showed a substantial decline in accuracy of GEBV over generations without retraining, since the selection candidates were more distant from the animals in the training population [25]. Although the decline in accuracy of GEBV was lower than expected based on the decline in genetic relationships and as observed in pedigree-based EBV, the average accuracy of GEBV in grand-progeny of training animals was similar to that of pedigree-based EBV in progeny and thus suggested a need for retraining every generation. Retraining resulted in increased accuracy of GEBV in subsequent generations [25] for all traits. Any decrease in the accuracy due to selection was outweighed by an increase in size of the training population.

### Impact on inbreeding and loss of alleles

Figure 2 clearly demonstrates the ability of GS to reduce rates of inbreeding. Sonesson and Meuwissen [28] observed even larger reductions in rates of inbreeding from GS because their conventional breeding program consisted of sib-testing for both sexes. The main reason for the reduction of rates of inbreeding with GS is that SNP genotypes provide information on Mendelian sampling terms, which reduces the emphasis placed on family information and, as a result, reduces correlations of EBV among family members and probabilities of co-selection of relatives, as demonstrated by [4]. This unique feature of marker genotype data, i.e., to increase accuracy of EBV and response to selection, while not increasing or even decreasing rates of inbreeding, is one of the main advantages of GS. Rates of inbreeding in GS programs will be greater if the selection candidates have strong genetic relationships with individuals in the training population because the information on relationships that is captured by genomic prediction will then result in greater correlations of EBV among selection candidates. This explains, in part, the greater rate of inbreeding for GS-all than for GS-1 and also the greater rate of inbreeding for GS-1 in the initial compared to later generations. Heidaritabar et al. [29] observed more directed and localized selection pressure on specific regions of the genome when using genomic information compared to pedigree-based selection, which agrees with our result that changes in allele frequencies had a greater range after implementation of genomic selection. The effects of such local inbreeding are not yet well understood but could represent signatures of selection as a result of large effect QTL.

## Conclusions

This study demonstrates the advantages of using genomic selection combined with cross-classified mating in layer chicken breeding programs, in terms of increased accuracy of prediction relative to pedigree-based parent average, providing the potential to shorten generation intervals and thereby increase response to selection per year. The advantages were reflected in the phenotypic superiority of the genomic sub-line for almost all traits included in the selection index. Genotyping also allowed sire assignment and relaxed traditional constraints on hierarchical matings. Using multiple sire matings improves the population structure and is expected to decrease inbreeding rate. Nevertheless, a sustainable breeding program still requires an effective population of sufficient size. In view of the rapid changes in genotyping technologies and costs, breeding programs based on genomic selection need to be re-evaluated and optimized on a regular basis.
